# The Action of Vitamin D in Adipose Tissue: Is There the Link between Vitamin D Deficiency and Adipose Tissue-Related Metabolic Disorders?

**DOI:** 10.3390/ijms23020956

**Published:** 2022-01-16

**Authors:** Izabela Szymczak-Pajor, Krystian Miazek, Anna Selmi, Aneta Balcerczyk, Agnieszka Śliwińska

**Affiliations:** 1 Department of Nucleic Acid Biochemistry, Medical University of Lodz, 251 Pomorska Str., 92-213 Lodz, Poland; agnieszka.sliwinska@umed.lodz.pl; 2 Institute of Applied Radiation Chemistry, Faculty of Chemistry, Lodz University of Technology, 15 Wroblewskiego, 93-590 Lodz, Poland; krystian.miazek@dokt.p.lodz.pl; 3 Department of Molecular Biophysics, University of Lodz, 141/143 Pomorska, 90-236 Lodz, Poland; anna.selmi@biol.uni.lodz.pl (A.S.); aneta.balcerczyk@biol.uni.lodz.pl (A.B.)

**Keywords:** vitamin D, adipogenesis, inflammation, lipid metabolism, oxidative stress

## Abstract

Adipose tissue plays an important role in systemic metabolism via the secretion of adipocytokines and storing and releasing energy. In obesity, adipose tissue becomes dysfunctional and characterized by hypertrophied adipocytes, increased inflammation, hypoxia, and decreased angiogenesis. Although adipose tissue is one of the major stores of vitamin D, its deficiency is detective in obese subjects. In the presented review, we show how vitamin D regulates numerous processes in adipose tissue and how their dysregulation leads to metabolic disorders. The molecular response to vitamin D in adipose tissue affects not only energy metabolism and adipokine and anti-inflammatory cytokine production via the regulation of gene expression but also genes participating in antioxidant defense, adipocytes differentiation, and apoptosis. Thus, its deficiency disturbs adipocytokines secretion, metabolism, lipid storage, adipogenesis, thermogenesis, the regulation of inflammation, and oxidative stress balance. Restoring the proper functionality of adipose tissue in overweight or obese subjects is of particular importance in order to reduce the risk of developing obesity-related complications, such as cardiovascular diseases and diabetes. Taking into account the results of experimental studies, it seemed that vitamin D may be a remedy for adipose tissue dysfunction, but the results of the clinical trials are not consistent, as some of them show improvement and others no effect of this vitamin on metabolic and insulin resistance parameters. Therefore, further studies are required to evaluate the beneficial effects of vitamin D, especially in overweight and obese subjects, due to the presence of a volumetric dilution of this vitamin among them.

## 1. Introduction

Adipose tissue is a loose connective tissue presented subcutaneously that surrounds organs and tissues. Apart from protecting internal organs against injuries, thermal isolation, and thermoregulation, adipose tissue is a key regulator of the energy supply and fat reservoir. It is composed of mature adipocytes, preadipocytes, mesenchymal stromal/stem cells (MSCs), vascular endothelial and contractile cells (smooth muscle cells and pericytes), and neurons, as well as various immune cells. Adipose tissue acts also as an endocrine organ due to the fact that it secretes numerous adipocytokines, such as hormones, pro- and anti-inflammatory cytokines, acute-phase proteins, and growth factors, that affect other cells and organs [1,2,3,4].

There are two types of adipose tissues: brown adipose tissue (BAT) and white adipose tissue (WAT). WAT, the most abundant type of adipose tissue containing adipocytes with large unilocular lipid droplets, plays a pivotal role in energy storage. WAT releases adipokines, i.e., adiponectin and leptin, that are involved in energy homeostasis [5]. In turn, adipocytes of BAT are characterized by the presence of many mitochondria and intracellular lipid droplets. Opposite to WAT, high levels of mitochondrial uncoupling protein 1 (UCP1) have also been observed in BAT. UCP1 is a molecule involved in thermogenesis, and it decreases the proton gradient via uncoupling the respiratory chain, as well as facilitates the synthesis of cyclic adenosine monophosphate (cAMP), leading to the promotion of thermogenesis capacity in mitochondria [6,7]. It has been reported that BAT also releases adipocytokines, including interleukin (IL)-6, which promotes insulin resistance. However, it is still not fully known whether BAT performs functions other than the regulation of thermogenesis [8,9,10].

There is also the third type of adipose tissue: beige/brite adipocytes that are immersed within the WAT and modifiable according to environmental factors, including diet and temperature. These two types of thermogenic fat cells possess a large number of mitochondria and express UCP1, presenting a high thermogenic potential and playing a crucial role in regulating systemic energy homeostasis in mammals [11]. Interestingly, the browning of WAT has also been proposed as a potential strategy to treat both obesity and obesity-related metabolic disorders. Notably, WAT transformation into a thermogenically active fat depot presents a promising therapeutic intervention in obese subjects with excess amounts of WAT [12].

Nowadays, 39% of teens and 13% of adults are overweight and obese, as documented in the 2020 World Health Organization report [3]. The overgrowth of WAT as a result of adipocyte hypertrophy and hyperplasia associated with overweightness and obesity is characterized by the altered release of adipocytokines [13,14]. Changes in the adipocytokine profile initiate a detrimental cascade of metabolic disturbances that trigger the development of insulin resistance, hyperglycemia, and dyslipidemia [1,13,14,15].

It has been suggested that overweightness and obesity coexist with vitamin D deficiency [16]. Deficiency and insufficiency are two expressions describing the status of vitamin D in the body below the optimal threshold. The best biomarker defining the vitamin D status is the concentration of 25-hydroxyvitamin D (25(OH)D; calcidiol), as the sum of the 25(OH)D_2_ and 25(OH)D_3_, in the serum [17]. According to the current classification, vitamin D deficiency is defined as serum 25(OH)D values below 20 ng/mL (50 nmol/L), and vitamin D insufficiency appears with serum 25(OH)D values between 20 and 30 ng/mL (50 and 75 nmol/L), while the optimal 25(OH)D level ranges between 30 and 50 ng/mL (75 and 125 nmol/L) [18,19]. Severe vitamin D deficiency corresponds to serum calcidiol concentrations below 12 ng/ml and increases the risk of excess mortality, immune system disorders, and many other diseases, whereas bone diseases such as rickets or osteomalacia but, also, fractures and bone loss already occur with calcidiol levels below 20 ng/mL [20]. Furthermore, vitamin D insufficiency/deficiency is also related to the development of metabolic diseases such as type 2 diabetes mellitus (T2DM) and non-alcoholic fatty liver disease (NAFLD). Taking into account multiple actions of vitamin D within the whole organism, including calcium homeostasis, vitamin D receptor (VDR)-mediated gene expression regulation, metabolism, immunomodulation, proliferation, and the differentiation of adipocytes and angiogenesis, it is not surprising that vitamin D deficiency affects the function of numerous tissues and organs, leading to the development of many diseases [21]. Thus, this review is focused on vitamin D actions in adipose tissue and the consequences of its deficiency leading to overweightness and obesity.

## 2. Methodology and Literature Search

To summarize the current scientific literature providing insight into the molecular mechanisms engaged in the molecular response of vitamin D in adipose tissue, PubMed, the Medical University of Lodz Library, and Google Scholar were searched to find the relevant articles published between 1967 and 2021. The following combinations of keywords were used: vitamin D OR vitamin D response OR vitamin D action OR genomic response of vitamin D OR non-genomic response of vitamin D OR vitamin D receptor OR genomic action of vitamin D OR non-genomic action of vitamin D OR molecular mechanism of vitamin D OR molecular response of vitamin D OR vitamin D insufficiency OR vitamin D deficiency OR vitamin D supplementation AND adipose tissue OR white adipose tissue OR brown adipose tissue OR lipid metabolism OR adipogenesis OR adipocytes OR adipocyte apoptosis OR adipocytokines OR adiponectin OR leptin OR lipid metabolism OR thermogenesis OR sub-inflammation OR oxidative stress OR immune cells OR reactive oxygen species OR immunomodulation OR overweight OR obesity OR NAFLD OR T2DM or metabolic syndrome.

## 3. Vitamin D Metabolism, Mechanism of Action, and Tissue Distribution

### 3.1. Metabolism and Molecular Response to Vitamin D

Vitamin D is a fat-soluble secosteroid. Its source is diet (mainly oily fish, mushrooms, and pharmaceutical supplements) or endogenous skin synthesis. In the skin, 7-dehydrocholesterol is transformed into pre-vitamin D under exposure to ultraviolet (UVB) light. Pre-vitamin D is immediately converted into vitamin D (cholecalciferol; calciol) as a result of a heat-dependent process. In the upper small intestine, dietary vitamin D is absorbed together with other dietary lipids as a result of passive diffusion [22,23,24]. Recently, it has been demonstrated that cholesterol transporters, including cluster of differentiation 36 (CD36), scavenger receptor class B type I (SR-BI), and Niemann–Pick C1-Like 1 (NPC1-L1), are also engaged in vitamin D transport across the enterocyte [25,26,27]. In enterocytes, vitamin D is loaded into chylomicrons and then released into the lymph nodes [28,29].

There are different vitamin D analogs, such as D_2_, D_3_, D_4_, and D_5_, present in various foods and produced from sterol precursors upon UV exposure, as depicted in Table 1. All vitamin D analogs are predicted to possess anti-osteoporotic, and calcium regulatory activities, as well as applications in hyperparathyroidism treatment [30]. Vitamin D_3_ is the main form of vitamin D in the diet of most people and is the one that is synthesized in the skin [31]. Although both D_3_ and D_2_ have vitamin D activity, some human studies have shown that D_3_ is more effective than D_2_, which shows less affinity for VDBP and hydroxylases [32,33,34].

**Figure 1:**
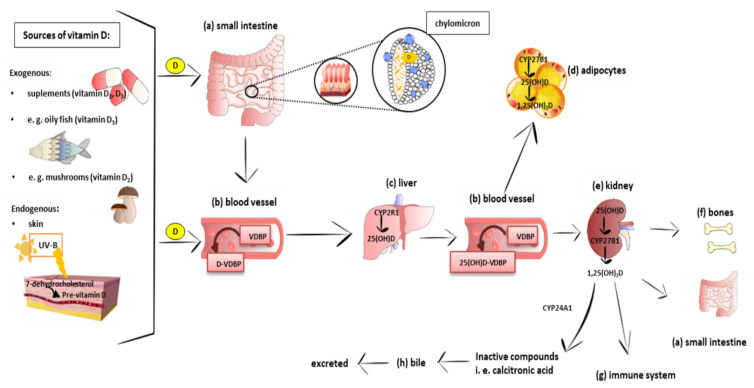
Schematic absorption, distribution, metabolism, and clearance of vitamin D indicating the strategic tissues/organs involved (**a**–**h**) and sources of the molecule. Main exogenous source of vitamin D are oily fish and mushrooms, apart from pharmaceutical supplements. The other source of the molecule is its synthesis in the epidermis layer of the skin. Under the influence of ultraviolet (UVB) radiation, 7-dehydrocholesterol undergoes nonenzymatic photoisomerization into pre-vitamin D. Next, pre-vitamin D is transformed into cholecalciferol in a heat-dependent process and comes out of the skin to the blood and binds to vitamin D-binding protein (VDBP) (**b**). Vitamin D from the diet is transported in blood mainly in chylomicrons (**a**). Next, both skin and diet-derived vitamin D reach the liver (**c**) and is hydroxylated into 25(OH)D (calcidiol) by 25-hydroxylase (CYP2R1). 25(OH)D leaves the liver and is bound to VDBP (**b**), which presents the highest affinity to calcidiol. 25(OH)D-VDBP is the major circulating form of vitamin D. Then, calcidiol is internalized by target tissues, mostly the kidneys (**e**), where it is transformed into the active form 1,25(OH)_2_D (calcitriol) by 1-hydroxylase (CYP27B1). CYP27B1 is expressed not only in the kidneys but also in numerous different cells, including adipocytes (**d**). The main function of vitamin D is to regulate calcium and phosphate homeostasis. The two main effector organs related to this function, on which the active metabolites of vitamin D act, are the intestines (**a**) and bones (**f**), and the molecule also exerts immunomodulatory effects (**g**). Calcitriol is able to stimulate its own degradation by the induction of 25(OH)D-24-hydroxylase (CYP24A1). CYP24A1 is an enzyme degrading both calcitriol and its precursor calcidiol to biological inactive metabolites, including calcitroic acid, which is excreted with bile (**h**). Abbreviations: D_2_, egrocalciferol, plant origin vitamin D; D_3_, cholecalciferol, animal origin food vitamin D; UVB, ultraviolet radiation; SR-B1, scavenger receptor class B type 1; CD36, class B scavenger receptor; NPC1-L1, Niemann-Pick C1-Like 1; CYP2R1, cytochrome P450 family 2 subfamily R member 1; CYP24A1, cytochrome P450 family 24 subfamily A member 1; CYP27B1, cytochrome P450 family 27 subfamily B member 1; CYP24A1, cytochrome P450 family 24 subfamily A member 1.

Calciol leaves the skin and is transported by vitamin D-binding protein (VDBP) in the blood. Vitamin D from the diet is transported into the bloodstream mainly in chylomicrons. After reaching the liver, both vitamin D of the diet and the synthesis of the skin are hydroxylated into calcidiol (25(OH)D) by 25-hydroxylase (CYP2R1). Next, calcidiol comes out of the liver to the blood and is transported by VDBP that possesses the highest affinity to calcidiol. Calcidiol-VDBP is known as major circulating form of vitamin D. Calcidiol reaches the target tissue, especially the kidneys, where it is metabolized into the active form of vitamin D, calcitriol (1,25(OH)_2_D), by 1-hydroxylase (CYP27B1). It has been reported that CYP2R1 [38] and CYP27B1 [39] are also expressed in 3T3L1 preadipocytes, subcutaneous adipose tissue (SAT), and visceral adipose tissue (VAT) in rats and humans [40]. Calcitriol is able to stimulate its own degradation by the activation of 25(OH)D-24-hydroxylase (CYP24A1). This enzyme is responsible for the degradation of both 1,25(OH)_2_D and its precursor 25(OH)D to inactive metabolites, i.e., calcitroic acid. Calcitroic acid is excreted with bile [41]. Calcitriol also promotes the formation and growth of bone by stimulating chondrocyte differentiation and elevating the serum phosphate and calcium levels [42]. An immunomodulatory role of vitamin D has been also proposed [43]. The schematic absorption, distribution, metabolism, and clearance of vitamin D is presented in Figure 1. Vitamin D, in order to exert a biological effect, binds to its receptor, vitamin D receptor (VDR), that is also found in 3T3-L1 adipocytes, [44] human pre-adipocytes, and differentiated adipocytes [45].

**Figure 2:**
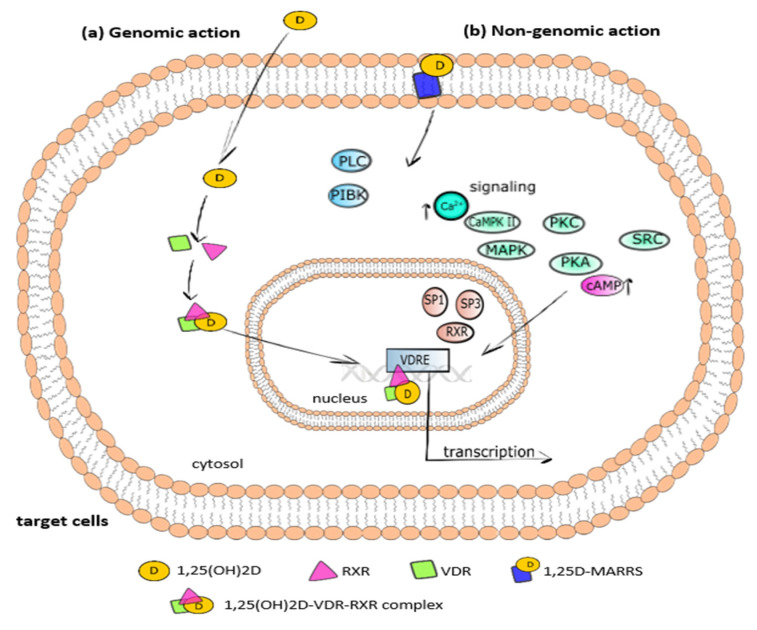
Summary of the cellular responses to vitamin D identified so far: (**a**) genomic and (**b**) non-genomic pathways. Active metabolites of vitamin D are characterized by a broad and diverse biological activity. In many tissues and cells, vitamin D binds to the nuclear vitamin D receptor (VDR) and then forms a heterodimer with the 9-cis retinoic acid receptor (RXR) with the properties of a transcription factor, which initiates (**a**) genomic action of the molecule. The nongenomic effects (**b**) are mediated by a membrane-located cellular receptor, which is distinct from the nuclear receptor and triggers intracellular metabolic pathways modulating the effects of gene expression. Abbreviations: D, 1,25(OH)_2_D; VDR, vitamin D receptor; RXR, retinoid X receptor; *VDRE*, vitamin D response element; 1,25D-MARRS, 1,25D-membrane-associated rapid response steroid-binding protein; PI3K, phosphatidyl-inositol-3 kinase; MAPK, mitogen-activated protein kinases; PLC, phospholipase C; CaMPKII, Ca^2+^-calmodulin protein kinase II; PKC, protein kinase C; Src, nonreceptor tyrosine kinase Src; PKA, protein kinase A; cAMP, cyclic adenosine monophosphate; SP1 and SP3, transcription factors. ↑ increase; ↓ decrease.

Physiologically, 1,25(OH)_2_D binds to VDR, which plays a role as a nuclear receptor and transcription factor. Vitamin D evokes genomic and nongenomic responses [46,47], as shown in Figure 2. In the genomic response after the interaction between 1,25(OH)_2_D and cytosolic VDR, VDR binds to the retinoid X receptor (RXR) (Figure 2a). The formed 1,25(OH)_2_D–VDR–RXR complex translocates from the cytoplasm to the nucleus and attaches to the vitamin D response element (*VDRE*) in the promoter region of vitamin D-dependent genes. Then, numerous enzymatic coregulatory complexes involved in the facilitation of histone epigenetic modification, the remodeling of chromatin, and the recruitment of local RNA polymerase II are mobilized and recruited. Finally, the expression of various vitamin D-dependent genes is regulated.

The nongenomic response to 1,25(OH)_2_D is activated via the binding of calcitriol with its membrane VDR, named the 1,25D-membrane-associated rapid response steroid-binding protein (1,25D-MARRS), as shown in Figure 2b. The interaction between 1,25(OH)_2_D and 1,25D-MARRS affects numerous cell signaling pathways via direct protein–protein interactions with various intracellular second messengers, such as cAMP, Ca^2+^, 3-phosphoinositides, and fatty acids [46,48], and kinases, i.e., phosphatidylinositol-3 kinase (PI3K), mitogen-activated protein kinases (MAPK)s, phospholipase C (PLC), Ca^2+^-calmodulin kinase II (CaMPKII), protein kinase C (PKC), src, and protein kinase A (PKA). Then, the signal is transduced to the transcription factors, including RXR, SP1, and SP3, that bind to VDRE on the promoter of vitamin D-regulated genes [21,49]. There is crosstalk between the genomic pathway activated by transcription factors and the nongenomic pathway activated by second messengers.

The list of genes whose expressions are regulated by vitamin D in the adipocytes, i.e., *IL-1*, *IL-6*, monocyte chemoattractant protein 1 (*MCP-1*), lipoprotein lipase (*LPL*), peroxisome proliferator-activated receptor γ (*PPARγ*), adipocyte-binding protein 2 (*AP2*), known as fatty acid-binding protein (*FABP4*), NADPH oxidase (*NOX*), transcription nuclear factor 2 (*Nrf2*), and thioredoxin (Trx), confirms the importance of this molecule for tissue and its ongoing metabolic processes [50,51,52,53,54].

As identified, on the one hand, vitamin D drives the metabolic pathways in adipose tissue, but on the other hand, the tissue maintains as a major storage of vitamin D [55] and, at the same time, a buffering system responsible for the slow release of the molecule to prevent the uncontrolled synthesis of its active form: 1,25(OH)_2_D. Moreover, vitamin D-metabolizing enzymes, including 25-hydroxylases (CYP2R1 and others less specific, e.g., CYP27A1 and CYP2J2), CYP27B1, and catabolic CYP24A1, are expressed not only in the liver and kidneys but also in adipose tissues [40,56,57,58]. The downregulation of CYP2J2 and CYP27B1 was observed in the adipose tissue of obese subjects [40]. It was found that VAT expresses higher levels of CYP27A1 but lower levels of CYP27B1 and CPY2J2 as compared to SAT [40]. Thus, SAT possess a lower potential to form 1,25(OH)_2_D. Furthermore,1,25(OH)_2_D upregulates VDR in VAT from obese subjects but not from lean subjects [57]. The presented evidence suggests that the activation of vitamin D and its actions in adipose tissues may differ depending on the degree of obesity and adipose depots.

### 3.2. Distribution of Vitamin D

As stated above, multiple organs and tissues are involved in vitamin D metabolism and signaling (Figure 1) [45,46]. A molecular distribution analysis performed in rodents and also in pigs showed different levels of the vitamin, depending on the tissue. A study on rats showed that supplementation with 5 µg of vitamin D (isotope ^14^C labeled) per day for 12 days resulted in the highest concentration of vitamin D in the kidneys, followed by adipose tissue, blood, and liver, and 3 weeks after the supplementation, the level of unaltered vitamin D_3_, its polar metabolites, and esters disappeared rapidly from most of the tissues but not from adipose, where pretty much its constant level was still detected after 80 days [55]. These data clearly pointed to fat as a vitamin D_3_ storage site what was later confirmed by Heaney et al. [59]. A high-performance liquid chromatographic analysis of tissue samples from pigs fed daily with approximately 2000 IU of vitamin D (cholecalciferol) showed that nearly 75% of cholecalciferol was accumulated in the fat. However, 25(OH)D was more evenly uniformly distributed all over the body as follows: 35% in fat, 30% in serum, 20% in muscle, and 15% in all other tissues. Taken together, vitamin D has been found to be predominantly stored in adipose tissue as cholecalciferol (vitamin D_3_) [59]. In turn, Piccolo et al. in their clinical trial found that a 13% loss of total body fat after 12 weeks of caloric restriction did not markedly exert an effect on the 25(OH)D level in SAT and the serum [60]. A long-term supplementation with 20,000 IU of vitamin D/week for 3–5 years revealed that the median vitamin D_3_ level in the abdominal SAT was more than six times higher in the patients supplemented with vitamin D as compared to the placebo group. Additionally, a higher level of 25(OH)D in the serum and SAT was observed in the vitamin D-supplemented group than in the placebo group. The obtained results showed that a large amount of vitamin D is stored in SAT. All patients participating in this study were characterized by an impaired glucose tolerance [61]. In-line with the above findings, Malmberg et al. [62] demonstrated that vitamin D and its metabolites are placed in adipocyte lipid droplets. Thus, adipose tissue is considered as a major vitamin D storage organ [63,64,65].

There is mounting evidence that the storage of vitamin D in adipose tissue is considered one of the major factors responsible for the comorbidity of vitamin D deficiency and obesity. Notably, vitamin D deficiency is often detected in obese individuals. In addition, it was observed that the serum 25(OH)D level declined with the increasing of the body mass index and body fat mass [66,67,68]. The results of a meta-analysis [69] revealed that the prevalence of vitamin D deficiency was about 35% and 24% higher in obese and overweight people, respectively, compared to those with normal body weights. Obese subjects exposed to UVB light or supplemented with 50,000 IU vitamin D presented a lower increase of the serum 25(OH)D level than normal body weight subjects [70]. Another studies also showed that the total amount of vitamin D (cholecalciferol) in the adipose tissue of obese humans and mice was higher compared to the normal body weight controls [71]. Along with these observations, it was suggested that vitamin D storage in body fat may reduce its bioavailability [70]. To explain these discrepancies, a “volumetric dilution” approach has been proposed [66], which allows the inclusion of body size in assessing the distribution of vitamin D in the serum and adipose fat. Accordingly, as the body size increases, the dilution of vitamin D in adipose tissue increases, and so, its serum level serum decreases. Thus, “volumetric dilution” is the reason for a decreased serum 25(OH)D level, as well as vitamin D deficiency, in obese subjects [71].

## 4. Vitamin D Action in Adipose Tissue

The growing body of evidence has revealed that vitamin D via its molecular mechanism of action is involved in numerous processes in adipose tissue, including adipogenesis, adipocyte apoptosis, metabolism of lipids, thermogenesis, and inflammation.

### 4.1. A Brief Insight into the Adipogenesis Process

**Figure 3:**
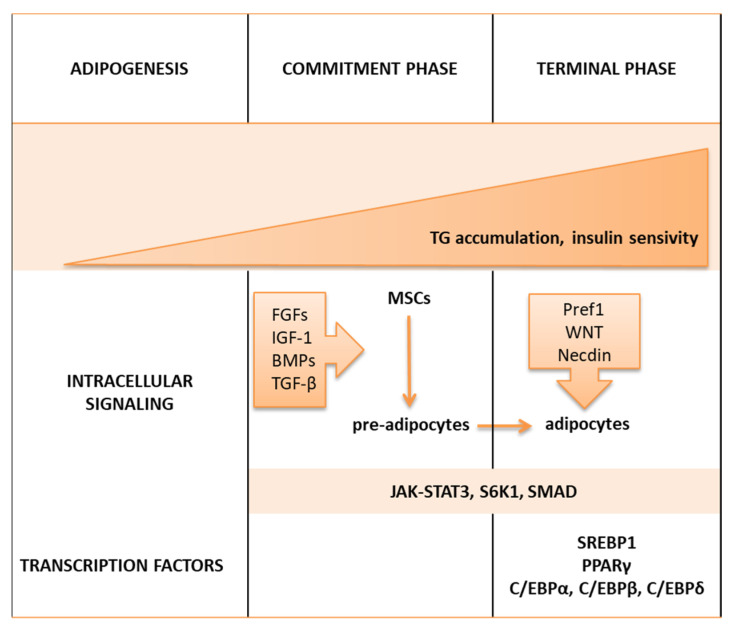
Intracellular molecules and transcription factors involved in adipogenesis. The figure shows the potential molecular mechanism of the adipogenesis-identifying intracellular-signaling network involved. Mesenchymal stem cells (MSCs) subjected to multiple growth factors are transformed into preadipocytes (commitment phase) that, in further steps, are stimulated to also form mature adipocytes with the support of transcription factors (terminal phase). Adipose tissue is considered to be the main storehouse of vitamin D. Due to the abundant expression of VDR in adipocytes, vitamin D affects the expression of multiple genes in these cells also related to adipogenesis. Moreover, vitamin D affects a number of processes, such as apoptosis and inflammatory processes, as well as redox homeostasis and oxidative stress. Abbreviations: JAK-STAT3, Janus kinase-signal transducer and activator of transcription 3; S6K1, ribosomal protein S6 kinase 1; SMAD, SMAD proteins; SREBP1, sterol regulatory-binding protein 1; C/EBPα, β, and δ, CCAAT/enhancer-binding proteins α, β, and δ; PPARγ, peroxisome proliferator-activated receptor γ; Pref1, preadipocyte factor 1; WNT, members of the wingless family; Necdin, melanoma-associated antigen family of proteins; Rb, retinoblastoma protein; FGFs, fibroblast growth factors; IGF-1, insulin-like growth factor 1; BMPs, bone morphogenetic proteins; TGF-β, transforming growth factor β.

Adipogenesis is a two-step process of the formation of mature adipocytes from mesenchymal stem cells (MSCs) regulated by multiple intracellular signaling molecules, i.e., Janus kinase-signal transducer and activator of transcription 3 (JAK-STAT3) [72], ribosomal protein S6 kinase 1 (S6K1) [73], SMAD proteins [74], and the induction of adipogenic transcription factors, i.e., sterol regulatory binding protein 1 (SREBP1) and PPARγ, as schematically shown in Figure 3.

In the commitment phase, several intracellular signaling molecules, i.e., fibroblast growth factors (FGFs), insulin like growth factor 1 (IGF-1), bone morphogenetic proteins (BMPs), and transforming-growth factor β (TGF-β), induce MSCs to form preadipocytes [75]. To proceed, further changes of preadipocytes, differentiation factors, are required to be secreted from their suppressive signaling molecules, including preadipocyte factor 1 (Pref1) [76], members of the wingless (WNT) family [77], Necdin, a member of the melanoma-associated antigen family of proteins [78], and proteins of the retinoblastoma (Rb) family [79].

In the second step, the terminal (adipogenic) phase, preadipocytes are differentiated into mature adipocytes, which undergo growth arrest, accumulating lipids and becoming insulin-responsive cells [80,81]. The terminal step of adipogenesis is regulated by various transcription factors, i.e., CAAT/enhancer-binding proteins (C/EBPβ, followed by C/EBPα and C/EBPδ), SREBP1, and PPARγ [82,83]. The terminal cascade begins with the early and transient expression of CCAAT/enhancer-binding protein (C/EBP) β and δ, leading to the activation of nuclear receptor PPARγ and C/EBPα. PPARγ and C/EBPα are critical transcriptional regulators of adipogenesis, followed by the accumulation of triglycerides (TG) [84]. AP2 is a carrier protein of fatty acids secreted by macrophages and adipose tissue that translocates ligands to the nuclear receptor of PPARγ and strengthens its transcriptional activity [85]. Transcription factors, including PPARγ and C/EBPα, activate the expression of numerous genes involved in insulin sensitivity, lipolysis, and lipogenesis, i.e., glucose transporter (GLUT-4), fatty acid synthase (FAS), AP2, and LPL [86,87,88].

### 4.2. The Action of Vitamin D in the Process of Adipogenesis—In Vitro Studies

Numerous studies have revealed that 1,25(OH)_2_D strengthens the differentiation of human and mouse adipose tissue-derived stem cells (ASCs) [50,58,89], as well as bone marrow-derived mesenchymal stem cells (BM-MSCs), from pigs [51] and mice [50] to adipocytes. Nimitphong et al. observed that 25(OH)D elevates the expression of CYP24A1, a primary target of nuclear VDR, and promotes adipogenesis in human ASCs, showing that the cells may generate the active form of vitamin D from 25(OH)D. Accordingly, with these findings, 1,25(OH)_2_D was detected in the culture media during the incubation of human ASCs with 25(OH)D [42]. Rat BM-MSCs stimulated with calcitriol (10 nmol/L) for 14 days exhibited an increased expression of AP2 and were differentiated into adipocytes [90]. Additionally, calcitriol increased the cell number about 180% in comparison to the untreated stromal cells [90], presenting a stimulatory effect on proliferation. It has been also observed that marrow stromal cells from rat fetuses in response to calcitriol (1 nmol/L – 1 µmol/L, for 3 days) undergo differentiation into adipocytes. The strongest effects were observed at a 100 nmol/L dose of vitamin D. Interestingly, vitamin D was only effective in the stimulation of the adipogenesis process when added during the initial growth stage (1–5 days) [91]. It has also been found that calcitriol stimulated both proliferation and differentiation toward the adipocytic phenotype via the elevation of PPARγ, AP2, and LPL at the mRNA level in porcine MSCs [51]. Pig BMSCs treated with calcitriol (either 10 nmol/L or 100 nmol/L) for 12 days decreased in proliferation but, at the same time, increased the lipid accumulation as compared to untreated cells. The upregulation of LPL, PPARγ, and AP2 in a dose-dependent manner was also observed [51]. Narvaes et al. also noted that the stimulation of MSCs with 1,25(OH)_2_D promotes differentiation toward adipocytes with increased lipid accumulation and the upregulation of adipogenic marker genes, including *FAS*, *PPARγ*, and *AP2* [50]. The stimulatory effect of calcitriol on the adipogenesis commitment phase have also been observed in BM-MSC derived from the wild-type but not from VDR knockout mice. Furthermore, re-transfection of the VDR knockout cells with human VDR rescued the pro-adipogenic activity of calcitriol [50]. However, whether calcitriol activates adipogenesis in human and mouse ASCs/MSCs via VDR-dependent mechanisms has not been shown yet.

Several studies have shown that calcitriol inhibits adipogenesis in BM-MSCs. Cianferotti and Demay reported that VDR^−/−^ mouse BM-MSCs activated for differentiation into adipocytes presented an increased expression of PPARγ, AP2, LPL, adipsin, and secreted fizzled-related protein 2 (SFRP2) and Dickkopf1 (DKK1) [92]. DKK1 and SFRP2 are responsible for the downregulation of WNT signaling via suppressing interactions with lipoprotein receptor-associated protein receptors (LRPRs), leading to a pro-adipogenic effect [93]. It has been demonstrated that calcitriol suppressed the transformation of mouse BM-MSCs into adipocytes by the inhibition of the SFRP2 and DKK1 expression levels via VDR-mediated WNT signaling [92]. Primary mouse BM-MSCs also presented a reduced expression of adipsin and AP2 in response to calcitriol [94,95]. Reduced lipid accumulation was presented in response to calcitriol in ST2 and PA6 cells [96,97]. In turn, Kelly et al. revealed that calcitriol significantly suppressed glucocorticoid- or thiazolidinedione-stimulated adipogenesis and inhibited the expression of adipsin and AP2 without an effect on LPL expression in BMS2 cells [95].

Extensive research has shown that vitamin D inhibits the terminal phase of adipogenesis [58,98,99,100,101,102,103,104,105] and reduces the lipid accumulation [100,101,104,106,107] in a 3T3-L1 pre-adipocyte cell culture. Several mechanisms responsible for the inhibitory effect of calcitriol on adipogenesis have been suggested. It was demonstrated that vitamin D inhibits adipogenesis via the downregulation of C/EBPα, PPARγ [98,99,100,101,103,104,107,108], C/EBPβ [99], and AP2 [58,98,100]. Kong and Li investigated the adipogenesis of VDR^+/+^ and VDR^−/−^ mouse embryonic fibroblasts. They found that liganded nuclear VDR (bound to calcitriol) is engaged in the suppression of the early phase of adipogenesis [98]. In contrast, the si-RNA-mediated knockdown of VDR in 3T3-L1 cells suppressed adipogenesis [109]. VDR expression was observed during the first 4 h of treatment of 3T3-L1 cells with dexamethasone, 3-isobutyl-1-methylxanthine, and insulin. Thus, VDR expression was found in the earliest stages of 3T3-L1 adipocyte differentiation and decreased during adipocyte maturation [108,109]. In turn, Blumberg et al. observed that calcitriol stimulates the expression of myeloid translocation proteins eight twenty-one (ETO)/MTG8, involved in carrying chromosomal translocation that results in a form of acute myeloid leukemia. ETO is known as a C/EBPβ corepressor [110]; thus, its activation inhibits the action of any C/EBPβ transcriptome effects needed for the terminal phase of adipogenesis [99]. As a result of ETO action, WNT10B expression is maintained, which, in turn, inhibits PPARγ expression [103]. WNT10B is a member of the WNT family and is expressed in adipose tissue. The WNT family operates through two signaling pathways: canonical and noncanonical [111]. The canonical pathway prevents the ubiquitination of β-catenin that translocates to the nucleus and activates the transcription of genes, i.e., *PPARδ*, which suppresses *PPARγ* expression [112]. Thus, WNT/β-catenin is responsible for maintaining the pre-adipocytes in an undifferentiated state, triggering the prevention of adipogenesis [77]. In 3T3-L1 pre-adipocytes, C/EBPβ has been shown to control WNT10B expression, which inhibits the antiadipogenic activity of WNT-β-catenin signaling [113]. The antiadipogenic effect of calcitriol is related to the maintenance of the expression levels of WNT10B and β-catenin in 3T3-L1 pre-adipocytes, which, in turn, contributes to the inhibition of PPARγ [103]. Additionally, calcitriol suppressed both the phosphorylation and mRNA expression of extracellular-regulated kinase (ERK). ERK is one of the MAPK-signaling molecules that inhibit adipocyte differentiation [101]. Similarly, a decreased lipid accumulation and reduced expression of *PPARγ*, *RXRA*, and other adipogenesis-related genes were also observed in human breast adipocytes [114] and primary porcine pre-adipocytes [115] in response to calcitriol. The inhibition of the differentiation of human mammary pre-adipocytes was observed not only after treatment with calcitriol but also after calcidiol [114].

Insulin promotes the accumulation of TG in adipocytes by increasing the glucose uptake and lipogenesis, activating the differentiation of pre-adipocytes to adipocytes and inhibiting lipolysis [116,117]. Some studies have also shown that calcitriol exerts an effect on the accumulation of TG in 3T3-L1 cells [101,102,103,104,106,107,118]. Most studies have shown that lipid accumulation decreases with increasing the calcitriol dose. Calcitriol reduced the lipid accumulation by the downregulation of SREBP1c and LPL [98,103]. SREBP1c activates the expression of transcription factors that induce the expression of genes engaged in lipogenesis, glucose metabolism, and the production of fatty acids [119,120]. VDRE has been identified in the promoter region of the *Insig2a* gene, which encodes a protein responsible for the blocking of SREBPs processing. It has therefore been suggested that calcitriol may regulate the expression of the SREBP1c by Insig2 [121]. Moreover, it was found that C/EBPα and C/EBPβ activate SREBP1c expression [82]; therefore, vitamin D may also regulate SREBP1c via their inhibition. LPL is engaged in TG hydrolysis from the movements of the lipoproteins and lipids. Additionally, LPL is known as a marker of adipocyte differentiation and increases TG accumulation [122]. On the other hand, calcitriol inhibits the expression of LPL, triggering a decreased lipid accumulation in 3T3-L1 cells [98,103]. In combination with genistein, calcitriol inhibits AP2 expression and fat accumulation in 3T3-L1 pre-adipocytes [100]. In mature adipocytes from porcine SAT, the downregulation of adipogenic genes, including phosphoenolpyruvate carboxykinase, steroyl-CoA desaturase, LPL, glycerol-3-phosphate dehydrogenase (*GPDH*), and *GLUT-4*, was observed in response to vitamin D. *GPDH* is expressed in mature adipocytes and known as a marker of adipogenesis. Its activity was markedly reduced in response to calcitriol. Additionally, a lower accumulation of lipids was demonstrated in porcine adipocytes [115].

Contrary to the above findings, some studies on human pre-adipocytes have revealed that vitamin D promoted the terminal phase of adipogenesis [50,51,58,89]. Both calcidiol and calcitriol strengthened adipogenesis in human and mouse pre-adipocytes by upregulating *PPARγ*, *LPL, AP2*, and *SREBP* and increasing TG accumulation [58]. Only one study showed that low doses of vitamin D (10 nmol/L) activated the accumulation of TG; however, higher doses (1 µmol/L) inhibited TG accumulation [102]. It has been also suggested that VDR itself may exert an influence on adipogenesis. Interestingly, unliganded VDR has been demonstrated to activate adipogenesis through increased lipid accumulation in 3T3-L1 cells [99].

Human pre-adipocytes were treated with calcitriol (100 nmol/L–100 pmol/L) to differentiate into mature adipocytes. A three-day calcitriol treatment increased the adipocyte differentiation and lipid accumulation. Moreover, calcitriol did not change the expression of C/EBPβ in human SAT but increased the C/EBPα. Stimulation with calcitriol during the late stages of differentiation increased the expression of LPL, AP2, and adiponectin. It is well-known that calcitriol exerts its genomic effects through VDR, the expression of which appears to be stable at the differentiation stage, but the level of proteins after differentiation is decreased [58]. The presented findings suggests that calcitriol regulates the expression of adipogenic factors during differentiation in adipose tissue. Sun et al. conducted a microarray study using human SAT pre-adipocytes derived from overweight females that were stimulated with calcitriol (10 nmol/L). The obtained results showed that calcitriol affected the genes engaged in the reduction of adipocyte apoptosis and increased the proliferation of adipocytes [123].

Gharbi-Chihi et al. observed an interplay between vitamin D and thyroid hormones in the terminal phase of adipogenesis in the pre-adipocytes of genetically obese mice. Triiodothyronine (T_3_) is a hormone involved in the terminal differentiation of mouse OB17 pre-adipocytes and the decreased expression of its own receptor [124]. In OB17 pre-adipocytes, calcitriol inhibited the T_3_ receptor sites at all stages of adipogenesis without affecting the affinity of T_3_ to its receptor. In this study, calcitriol in a concentration below 250 pmol/L showed a stimulatory effect on adipogenesis, whereas, above 250 pmol/L, an inhibitory effect [125].

Taken together, the results of the studies based on the effect of vitamin D on adipogenesis present inconclusive results. Thus, more studies are required to investigate the effects of vitamin D separately on the commitment and terminal phase of adipogenesis.

### 4.3. The Effect of Vitamin D in Adipogenesis—Animal Models

The most-studied animal models analyze the role of vitamin D in the regulation of adipogenesis, as well as vitamin D supplementation on the body composition in adipocyte–VDR null mice. Deletion of the adipose tissue-specific VDR resulted in changes in the size/obesity of the animals, with gender-specific effects: females, but not males, showed increased visceral fat mass; nevertheless, both presented the lean phenotype [126]. It has been also observed that a lack of VDR triggered a pro-adipogenic effect of calcitriol action. VDR^−/−^ mice showed an elevated food intake [127,128] and lower adipose tissue mass with the same amount of BAT [127], lower serum TG, and cholesterol [128,129] in comparison to wild-type individuals. Detailed metabolic analyses suggested that fat in VDR^−/−^ mice is a main energy source, contributing to their leaner phenotype. The WAT of VDR^−/−^ mice characterized a 25-fold increased expression of UCP1, which is involved in heat production by thermogenesis [127].

On the other hand, mice with overexpressed VDR in adipose tissue presented the obese phenotype and reduced energy metabolism [127,128,129,130]. Reduced fatty acid oxidation and lipolysis were also observed in the adipocytes of VDR^+^ mice, as well as the downregulation of key genes such as adipose triglyceride lipase (ATGL) and hormone-sensitive lipase (HSL) regulating these processes. The obtained results have also shown that increased storage and reduced fat utilization leads to the obese phenotype [131]. Those data are in-line with the findings from other studies showing that the primary culture of adipose cells from VDR^+^ mice stimulated with calcitriol triggers leptin production in a VDR-dependent manner [130]. Narvaez et al. revealed that the lean phenotype and defective cellular adipogenesis in VDR null mice become apparent with age [127]. Moreover, a decreased adipocyte size was shown in older (1 year), but not young (21 days), mice [132]. In contrast, maternal vitamin D deficiency had no effect on adipose tissue development in the offspring [132,133]. The presented results suggest that a lack of VDR does not affect fat deposition in the early stages of development. It has been also proposed that the age-related lean phenotype in VDR^−/−^ mice may result from a reduction of energy expenditure by calcitriol–VDR during the uncoupling process [134].

The study on mice fed with a AIN93D diet, which contained 1 IU of calcidiol (0.025 µg/kg; standard conditions), or a AIN93G diet containing 10 IU of the compound (0.25 µg/kg) for 3 weeks, showed an increase in SAT and VAT in mice fed with the AIN93G diet in comparison to the control individuals [135]. The continuous dose of calcitriol (18 pmol/d) delivered by minipumps to SAM-P/6 mice for 6 weeks markedly reduced the adiposity and PPARγ mRNA expression, presenting a mechanism by which calcitriol blocks adipogenesis [136].

It has been also presented that calcitriol and calcium work together to influence the energy metabolism and adipogenesis. Sergeev et al. observed that mice fed a high-fat diet with calcitriol and calcium presented lower body weights and reduced weight gains, and total WAT compared to mice with diet-induced obesity on the standard chow (0.6% calcium, 0.025 μg/kg calcitriol). Calcitriol and calcium both separately and in combination markedly elevated WAT apoptosis. It has been also proposed that the vitamin D-dependent induction of Ca^2+^-mediated apoptosis pathways is responsible for the antiadipogenic effect of the supplemented diets [137].

Summarizing, the results of studies focused on the effect of vitamin D on the terminal phase of adipogenesis are still inconclusive. Further molecular studies are required to better understand the influence of vitamin D on the key molecules involved in adipogenesis, including C/EBPα, C/EBPβ, C/EBPδ, and PPARγ.

### 4.4. Vitamin D and Apoptosis of Adipocytes

Detailed analyses of adipocytes metabolism revealed the role of vitamin D in apoptosis. Gene profiling performed by a microarray on human subcutaneous adipocytes from obese subjects showed the antiapoptotic potential of calcitriol [123] that, in further studies, pointed at UCP2 being involved in the whole process [138]. Zemel and Sun proposed that low doses of vitamin D elevate the mitochondrial potential and ATP yield, inhibiting UCP2 and leading to the suppression of apoptosis [139]. UCP2 is highly expressed in WAT and acts as a mitochondrial uncoupler of oxidative phosphorylation, leading to the decreasing efficiency of ATP synthesis. Thus, it has been also proposed that UCP2 may stimulate apoptosis in adipocytes [140]. Interestingly, it was identified that the effect of calcitriol on apoptosis is concentration-dependent; high doses stimulate the process, whereas low ones inhibit it [140]. In turn, an increased calcitriol intake seems to be negatively associated with WAT weight due to the activation of apoptosis, as identified in a murine model of obesity induced by a high-fat diet (HFD) [137].

The mechanism responsible for the induction of apoptosis by vitamin D has been shown to be closely related to the level of calcium, which vitamin D affects [138,141,142]. As identified, apoptosis is activated by a vitamin D-induced Ca^2+^ influx from the extracellular space and activation of the Ca^2+^ release from the endoplasmic reticulum stores via voltage-insensitive Ca^2+^ channels (VICC), the ryanodine receptor/Ca^2+^ release channels (RyR), and the InsP_3_ receptor/Ca^2+^ release channel (InsP_3_R). Subsequently, the activation of μ-calpain by sustained cytosolic Ca^2+^ signaling triggers the stimulation of Ca^2+^/calpain-dependent caspase-12 [138,141,143].

Taken together, little research has been carried out on the effects of vitamin D on adipocyte apoptosis. The results of the studies conducted so far are inconclusive and suggest the influence of vitamin D on UCP and calcium metabolism. Therefore, an interesting problem seems to be evaluation of the impact of vitamin D on the apoptosis of hypertrophied and dysfunctional adipocytes, especially visceral WAT, with infiltrates of inflammatory cells from obese subjects.

### 4.5. Vitamin D as a Regulator of Metabolism and Adipocytokines Secretion in Adipose Tissue

#### 4.5.1. Vitamin D as a Regulator of Lipid Metabolism in Adipose Tissue

Lipid metabolism, including lipolysis and lipogenesis, is a complex process that is differentially regulated not only in BAT and WAT but also in visceral and subcutaneous WAT. In vitro and in vivo studies suggest that the effect of vitamin D on lipid metabolism is closely related with the type and location of adipose tissue.

Namely, vitamin D was found to stimulate lipogenesis in subcutaneous WAT, whereas it inhibited this process in visceral WAT. It has been documented that calcitriol stimulates the activity of FAS and increases the FAS protein level in human subcutaneous adipocytes. This effect was mimicked by membrane VDR agonists and inhibited by membrane antagonists [144]. Thus, this suggests that the calcitriol-dependent increase of FAS is VDR-mediated [50,134]. Moreover, the increased mRNA level of LPL, the protein level of AP2, and increased TG accumulation were found after the calcitriol stimulation of differentiated subcutaneous human adipocytes isolated from male and female donors with 25.6–50.9 kg/m^2^ BMI [58]. Interestingly, Kang et al. determined the effect of vitamin D on the expression of genes involved in lipogenesis in abdominal adipose tissue and the liver. Vitamin D was administered in a dose of 40 IU (1 μg/kg/day) to pregnant Sprague–Dawley rats. The obtained results showed the downregulation of lipogenesis-associated gene expression, i.e. *FAS*, *SCD1*, and *ACC1* in both abdominal adipose tissue and the liver in response to vitamin D. The presented effects contributed to the suppression of fat synthesis in abdominal adipose tissue and in the liver and the decreased body weight of pregnant rats [145].

Adipocyte lipolysis is under hormonal regulation. Numerous lipolytic hormones, i.e., catecholamines, act through β-adrenergic receptors to induce an increase in cAMP. In turn, cAMP activates cAMP-dependent PKA, which phosphorylates and activates lipolysis enzymes, including CPT1α, PGC1α, PPARα, UCP1, SIRT-1, LPL, and hormone-sensitive lipase (HSL) [146,147]. Similarly, to lipogenesis, the effect of vitamin D on lipolysis is suggested to be dependent on the WAT localization. It was found that calcitriol inhibited adipocyte basal lipolysis in a human subcutaneous adipocytes culture as a consequence of the elevation of the Ca^2+^ level and a decrease of cAMP and the resulting HSL phosphorylation [144]. In contrast, calcitriol increased the rate of lipolysis in murine 3T3-L1 adipocytes differentiated from mouse embryo fibroblasts, which was accompanied by a higher secretion of glycerol [147,148].

In turn, in the BAT of diet-induced obese mice, a vitamin D supplementation was demonstrated to increase the expression of genes such as *Pgc-1a*, *Pgc-1b*, *Cpt1b*, *Mcad*, *Lcad*, and *Pdk4* and thereby enhance the oxidation of fatty acids [149].

Taken together, the available studies have shown that the effect of vitamin D on lipolysis and lipogenesis is dependent on the type of adipose tissue and the origin of adipocytes (localization). Namely, vitamin D stimulates lipolysis in visceral adipose tissue but decreases this process in subcutaneous tissue. Likewise, vitamin D stimulates lipogenesis in subcutaneous tissue but inhibits it in visceral adipose tissue. In BAT, vitamin D increases fatty acid oxidation. It seems that the effect of vitamin D on lipid metabolism is a result of genomic and nongenomic responses to the vitamin.

As confirmed by multiple studies, the development of insulin resistance in adipocytes is associated with disturbed insulin signaling. In the insulin resistance state, adipose tissue is characterized by the decreased expression of IRS-1 and GLUT-4 and, at the same time, upregulation of IRS-2 [116,150,151,152]. The results of human studies have shown that vitamin D reduces insulin resistance indirectly by improving metabolic parameters such as HOMA-IR, a fasting plasma glucose. However, there is little research focused on the effects of vitamin D on the components of the insulin pathway in adipocytes. Manna et al. demonstrated that calcitriol reduced the insulin resistance by the improvement of the glucose uptake, which, in turn, is a result of the GLUT-4 translocation increase in the highly glucose-treated 3T3-L1 adipocytes and adipose tissue of HFD-fed diabetic mice [52]. Therefore, more molecular in vitro and in vivo research determining the effects of vitamin D on the components of the insulin signaling pathway (especially IRS and GLUT-4) is required.

#### 4.5.2. The Effect of Vitamin D on Production of Adipocytokines

A growing body of evidence has indicated that vitamin D is involved in the control of adipokine synthesis and secretion, including the major ones, leptin and adiponectin [130,153,154].

Adiponectin is commonly known as an insulin-sensitizing and anti-inflammatory hormone produced by adipose tissue and the brain [155,156,157,158]. This hormone not only increases the glucose uptake by skeletal muscle and reduces hepatic gluconeogenesis but also mobilizes fatty acid oxidation and glucose utilization in both skeletal muscle and the liver [159]. The biological activity of this hormone depends on its isoforms, serum concentration, and tissue-specific receptor subtype. A negative relationship between the body mass index (BMI) and circulating adiponectin level has been reported.

The downregulation of adiponectin, particularly the high molecular weight isoform, has been observed in obese children with a vitamin D deficiency [23,154]. In turn, an increased level of adiponectin has been found in T2DM patients supplemented with vitamin D-fortified food [160]. Walker et al. noted that calcitriol treatment elevates the expression of adiponectin and disulfide bond-A oxidoreductase-like protein (DsbA-L), which is involved in the multimerization of adiponectin [154]. It has been also shown that calcitriol treatment can upregulate adiponectin in vitro and inhibit anti-inflammatory cytokine expression and that a daily intake of fortified vitamin D can decrease inflammation in T2DM [160,161,162]. One study has shown that calcitriol did not alter adiponectin expression in a human adipocyte culture [163]. In turn, Dinca et al. showed that vitamin D supplementation had no effect on the adiponectin levels in overweight and obese subjects [164]. The results of another study documented not only the suppressed TNF-α-stimulated secretion of MCP-1 but also the inhibited secretion of adiponectin from differentiated adipocytes of subcutaneous VAT after supplementation with vitamin D and TNF-α [165].

Adipose tissue secretes leptin, which acts on the hypothalamus to suppress appetite and increase energy expenditure [166]. Leptin controls lipid metabolism by stimulating lipolysis and inhibiting lipogenesis [167,168]. Leptin synthesis is stimulated by insulin, TNF-α, estrogens, and glucocorticosteroids but inhibited by growth hormone and free fatty acids [169].

It was reported that vitamin D participates in both adipokine release and energetic homeostasis by controlling leptin production. It has been also shown that vitamin D inhibited the release of leptin by adipose tissue [170]. In vivo, CYP27B1 knockout mice exhibited hypoleptinemia symptoms when they were fed more food compared to their wild-type counterparts. In contrast, VDR knockout (VDRKO) mice presented the lean phenotype with concomitant hypoleptinemia and hyperphagia [127]. It has been also proposed that the adipose tissue mass reflected the level of leptin in the serum. However, it was not fully known whether hypoleptinemia is a consequence of the body fat content or the effect of the vitamin D/VDR system on leptin expression in VDRKO mice. It is known that calcitriol directly activates both the leptin release and expression in wild-type mouse adipose tissue cultures. However, the effect shown was not detected in VDR null mice adipose tissue cultures.

Furthermore, it was reported that calcitriol downregulates leptin by at least 84% in mouse 3T3-L1 adipocytes [171]. Interestingly, it has been also observed that leptin inhibits the renal transformation of calcidiol to calcitriol indirectly by the activation of osteoblasts and/or osteocyte secretion of a fibroblast growth factor (FGF-23) [172]. It is well-known that FGF-23 inhibits calcitriol synthesis by suppressing renal CYP27B1. Bouillon et al. observed a decrease of adipose tissue mass, a reduction of serum leptin levels, and a higher food intake in VDR knockout mice compared to wild-type mice [173]. A similar effect was noted by Narvaez et al. in mice unable to generate calcitriol due to a lack of CYP27B1 expression [127]. Overweight/obese adults with vitamin D deficiency who received vitamin D supplementation for 16 weeks showed a significant increase of leptin in the vitamin D group compared to the placebo group [174].The results of a meta-analysis including six clinical trials showed that vitamin D supplementation markedly elevated the serum leptin level [175].

Taken together, the results of the majority of the presented studies demonstrated that vitamin D exerted a significant impact on the adiponectin and leptin levels in obesity or diabetes and reduced the associated disorders, such as inflammation, energy homeostasis disturbances, and overgrowth of adipose tissue.

### 4.6. Effects of Vitamin D on Adipose Tissue Inflammation

Adipose tissue represents a unique collection of numerous immune cells that are involved in immune homeostasis [176]. Adipocyte hypertrophy results in an unbalanced blood flow, triggering necrotic cell death, local hypoxia, and inflammatory macrophage infiltration. As a consequence, overgrowth adipocytes secrete a reduced level of adiponectin and increased levels of MCP-1, IL-1β, IL-6, IL-8, and TNF-α [177,178,179,180,181,182]. Moreover, adipocytes and macrophages express Toll-like receptors (TLRs). Food lipids bind TLR2 and TLR4 on adipocytes and macrophages, which, in turn, activates the nuclear factor (NF)-κB-signaling pathway and stimulates the expression of inflammatory cytokines [183]. An increased expression of proinflammatory cytokines is involved in adipose tissue inflammation via the recruitment of monocytes/macrophages into adipose tissue and promoting them toward the proinflammatory M1 type [184]. The infiltration of other immune cells, i.e., neutrophils, natural killer (NK) cells, and CD8^+^ T cells, precedes macrophage accumulation and contributes to their activation [185]. It has been also estimated that macrophages account for more than 50% of immune cells in obese adipose tissue. In contrast, the adipose tissue of lean people shows that macrophages make up less than 10% of the cells of the immune system, presenting a M2 anti-inflammatory profile. The increased secretion of proinflammatory cytokines (i.e., IL-1β, IL-6, and MCP-1) by both adipocytes and macrophages has been observed in the adipose tissue of obese subjects. [186]. A growing body of evidence has shown that low-grade chronic adipose tissue inflammation is an important risk factor for the development of metabolic diseases, including T2DM. It has been demonstrated that a macrophage-conditioned medium can promote the secretion of proinflammatory factors, including IL-6, IL-8, MCP-1, and chemokine (C–C motif) ligand 5 (CCL-5) and numerous proteins involved in extracellular matrix remodeling by both human pre-adipocytes and adipocytes. Those secreted factors may stimulate inflammation, fibrosis, and insulin resistance in adipose tissue [187,188,189,190].

The effect of vitamin D on adipose inflammation has been also extensively investigated. VDR and CYP27B1 are expressed in both adipocytes and macrophages. Thus, both calcitriol or calcidiol may control the inflammatory responses in adipose tissue [191]. Interestingly, it has been documented that the mRNA expression of proinflammatory cytokines (IL-6, IL-8, MIF, and CD14) and the protein levels of IL-6, MIP, MCP-1, and M-CSF were increased in response to 10 nmol/L calcitriol in human adipocytes and differentiated 3T3-L1 cells [163,192]. Although these studies suggested a proinflammatory effect of vitamin D, the results of some studies have indicated its anti-inflammatory properties. Several studies have revealed that calcitriol downregulated numerous cytokines, including IL-1β, IL-6, IL-8, and MCP-1, in both pre-adipocytes and adipocytes [133,161,193,194,195,196]. It is known that vitamin D also presents anti-inflammatory activity in monocytes/macrophages, and several mechanisms are involved in these effects. The downregulation of both the protein and mRNA levels of TLR2 and TLR4 in human monocytes was identified due to calcitriol treatment in a time- and dose-dependent manner. As a consequence, the levels of IL-6 and TNF-α were also reduced [197,198]. However, this decrease of TLR2 and TLR4 in response to calcitriol was suppressed upon stimulation with a VDR antagonist. This confirms that the presented immunomodulatory effect of calcitriol on TLRs is VDR-dependent [198]. Park et al. also observed that TLR2 expression was also reduced by calcitriol in immune cells resident in adipose tissue [199]. 

**Figure 4:**
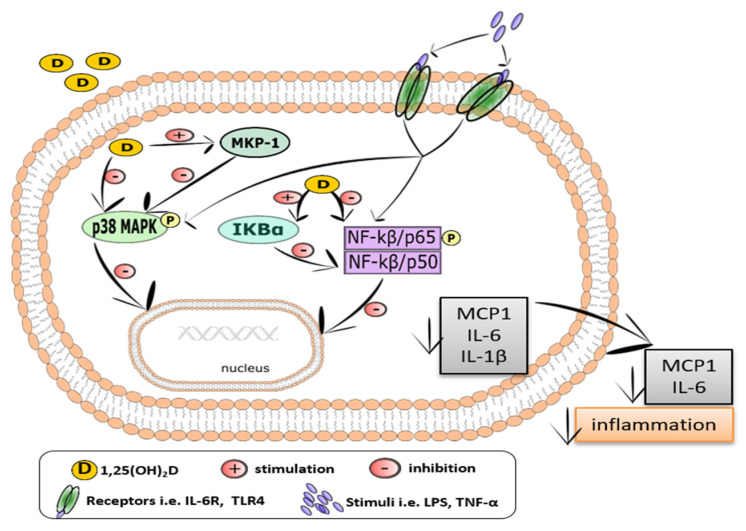
The effect of vitamin D on the MAPK and NF-kB pathways engaged in adipocyte inflammation. NF-κB is a key transcriptional factor involved in multiple processes, e.g., proliferation but also at the first step in the regulation of the inflammatory response due to controlling the release of antimicrobial molecules, including cytokines and chemokines, also in pre-adipocytes and adipocytes, where vitamin D was found as an inhibitor of the processes NF-κB- and MAPK-driven. Abbreviations: MKP-1, MAPK phosphatase-1; p38 MAPK, p38 mitogen-activated protein kinase; IKBα, nuclear factor-κappa B inhibitor alpha; MCP1, monocyte chemoattractant protein 1; IL-6, interleukin 6; IL-1β, interleukin 1β; LPS, lipopolysaccharide; TNF-α, tumor necrosis factor α; IL-6R, interleukin 6 receptor; TLR4, Toll-like receptor 4. ↑increase; ↓decrease.

Vitamin D was also found to suppress the TLR-mediated-signaling pathway, including NF-κB and MAPK [193,194,198,199,200]. The signaling of inflammatory pathways in adipose tissue engages the induction of NF-κB and translocation of p65 from the cytoplasm to nucleus, which is associated with the degradation of IκBα [201], a potent NF-κB inhibitor [193,194,198,199,200,202]. Mutt et al. [203] showed that calcitriol inhibited the LPS-induced secretion of IL-6 in human isolated mature and MSC-differentiated adipocytes. The presented action of calcitriol was also confirmed by Marcotorchino et al. [161], Gao et al. [202], and Ding et al. [204], who revealed that calcitriol reduced the inflammatory markers in both human and mouse adipocytes via participating in the inhibition of the p38 MAP kinase and NF-κB classical inflammatory pathways, as presented in Figure 4. Calcitriol inhibits NF-κB activation via blocking the cytoplasm to nucleus NF-κB/RelA translocation, increasing IκBα expression and decreasing p65 phosphorylation. The results from studies using human adipocytes and 3T3-L1 cells have also shown that calcitriol (at doses 10–100 nmol/L) inactivated NF-κB via the activation of IκBα and suppression of IL-6, MCP-1, and IL-1β production [161,202,204].

Vitamin D reduces the level of phosphorylated p38 MAPK and Erk 1/2, as well as increases the level of MAPK phosphatase-1 (MKP-1). In turn, MKP-1 inactivates MAPK signaling via the suppression of p38 activation. The presented effect of vitamin D has been shown both in pre-adipocytes and differentiated adipocytes [161,199,202,204]. A VDR-binding site has been identified in human, as well as mouse, MKP-1 promoters [195]. Both calcitriol and calcidiol have been reported to reduce LPS-induced TNF-α and IL-6 production via the suppression of p38 MAPK activation in human monocytes/macrophages [195].

Several in vitro studies have documented that calcitriol decreased chemokine and cytokine release by adipocytes and the chemotaxis of monocytes [161,165,202,204,205]. Lorente-Cebrian et al. proposed that calcitriol attenuates TNF-α-activated MCP-1 secretion [165]. Calcitriol has also been reported to reduce the expression of TNF-α, IL-1, IL-6, and IL-8 in peripheral blood mononuclear cells derived from T2DM patients [206]. Roy et al. noted that calcidiol and calcitriol reduced the secretion of cytokines from omental adipose tissue but not SAT. Interestingly, the presented effects occurred only in women, but not in men, adipose explants [153].

Some animal studies have also shown that vitamin D supplementation exerts an anti-inflammatory effect. Vitamin D supplementation not only reduce the secretion of proinflammatory cytokines (IL-6, IL-1β, and TNF-α) and chemokines (CCL2, CCL5, CXCL10, and CXCL11) but also modulate the recruitment of immune cells to adipose tissue [162,196,199,207,208]. Karkeni et al. observed that vitamin D decreased the levels of proinflammatory cytokines and chemokines in both diet-induced obese mice, an animal model of metabolic inflammation, and in mice injected with LPS, an animal model of acute inflammation. Furthermore, mice fed with HFD and supplemented with cholecalciferol at a dose of 3000 IU/kg (75 µg/kg) body weight showed a reduction in the number of T cells and M1-type macrophages in adipose tissue compared to mice fed with HFD and supplemented with vitamin D at a dose of 300 IU/kg (7.5 µg/kg) body weight [196]. Similarly, another study revealed that HFD-fed mice supplemented with calcitriol at a dose of 2000 IU/kg (50 µg/kg) decreased the IL-6 level in epididymal adipose tissue [162]. However, the results of studies focused on both the vitamin D level and the effect of vitamin D supplementation on the serum levels of inflammatory cytokines in human have provided inconclusive findings. It has been reported that, accordingly with the increase of the serum calcidiol concentration, the level of plasma IL-6 and TNF-α decreased in normal weight subjects [209]. Another study presented that vitamin D had no effect on the inflammatory biomarkers in both the serum and adipose tissue in obese subjects. It is worth emphasizing that this randomized controlled trial was conducted on a small number of participants: 40 subjects. Only 22 subjects received cholecalciferol in a dose of 7000 IU/day for 26 weeks, and 18 subjects were included in the placebo group [210]. Interestingly, Yu et al., as a result of a meta-analysis of randomized controlled trials, observed that vitamin D supplementation reduced the level of C-reactive protein (CRP) without affecting IL-6 and TNF-α. The studies included in a meta-analysis showed differences in study designs, the dose of supplemented vitamin D, and the duration of the supplementation [211]. The results from a meta-analysis carried out by Jamka et al. showed that vitamin D supplementation in overweight and obese patients did not affect the level of TNF-α, IL-6, and CRP. The number of studies included in the meta-analysis was small, with a limited number of participants. Moreover, the supplementation with vitamin D and calcium triggered an observed effect that was difficult to distinguish between those two components. Notably, the dose of vitamin D and the duration of the vitamin D supplementation were different and may have had an effect on the interpretation of the collected data. [212]. Undoubtedly, further studies are needed to determine the effect of vitamin D supplementation on the levels of inflammatory cytokines in humans.

Chronic activation of the immune cells can trigger inflammation and obesity-related pathogenesis, in which insulin resistance is involved. Two distinct mechanisms responsible for insulin resistance have been documented: the first is obesity-related insulin resistance, attributed to macrophage-driven inflammation [213], whereas the second mechanism is not associated with obesity but is age-related insulin resistance regulated by fat-resident regulatory T cells (fTreg) [214]. Therefore, immune changes in obesity and diseases. T2DM must be taken into consideration in studies of the effects of vitamin D on the immune system. Adipose-resident lymphocytes, i.e., invariant natural killer T (iNKT), might be depleted in obesity [215]. It has been also noted that, in obesity, the activity of T-lymphocytes is modulated [216]. Moreover, regulatory T cells (Treg) were significantly reduced in VAT in obese mice [217]. It has been proposed that vitamin D decreases inflammation by enhancing the suppressive activity of Tregs [217]. However, the influence of vitamin D on fTreg is still not fully known [218]. It has also been observed that vitamin D–VDR interactions inhibited the migration of monocytes into adipose tissue by downregulation of the production of chemokines from numerous cell types in mice [196]. Moreover, calcitriol occurred to regulate the function of macrophages and other immune cells in adipose tissue [219,220].

Summarizing the presented data, it may be concluded that vitamin D suppresses the secretion of proinflammatory cytokines, including IL-6, TNF-α, and C-reactive protein. Calcitriol markedly inhibits NF-κB and MAPK-signaling pathways, leading to the prevention of proinflammatory gene transcription, and thereby decreases inflammation in adipose tissue. The latter one is of special importance, since low-grade chronic inflammation accompanies obesity-related disorders, including insulin resistance, diabetes, and NAFLD.

### 4.7. Oxidative Stress in Adipose Tissue

The elevated level of oxidative stress in adipocytes and adipose tissue is ascribed to the development of metabolic disorders such as obesity and T2DM. The main sources of reactive oxygen species (ROS) generation in adipocytes/adipose tissue are mitochondria and NOX, as a consequence of an excessive influx of nutrient (sugars and fatty acids). Nutrients are utilized during cellular respiration, and ATP production occurs through the electron transport chain (ETC) in the oxidative phosphorylation system. The increased nutrient supply leads to the overproduction of NADH and the excess supply of electrons to the transport chain, which, with low ATP requirements and a low respiration rate, result in the leakage of O_2_^•−^ as a byproduct from complexes I and III in mitochondrial ETC. Oxidative stress can be also elevated via NADPH oxidase 4 (NOX4), generating H_2_O_2_, as an over-nutrient supply results in the upregulation of NOX4 expression, leading to increased ROS production in adipocytes. Oxidative stress developed in adipocytes/adipose tissue can further contribute to their dysfunction, with implications for the whole organism [221,222,223].

Oxidative stress can be defined as a disturbed balance between ROS production and the antioxidant defense system, characterized by the ROS level exceeding the capacity of the antioxidants enzymes (superoxide dismutase (SOD), catalase (CAT), and glutathione peroxidase (GPx)) and low-molecular-weight antioxidants (glutathione (GSH), vitamin C, vitamin E, and β-carotene) to maintain the oxidative balance. Various ROS, such as superoxide anion (O_2_^•−^), hydroxyl radical (^•^OH), and hydrogen peroxide (H_2_O_2_), can damage cellular macromolecules. The exposure of lipids, proteins, and nucleic acids to ROS leads to oxidative damage, including lipid peroxidation (i.e., malondialdehyde, MDA; 4-hydroxynonenal, 4-HNE; F2-isoprostanes; F2-IsoPs); protein oxidation (protein carbonyl); and nucleic acid oxidation (8-hydroxyguanine) products [221,222,223,224].

Oxidative stress and inflammation are two interrelated conditions characterizing the pathophysiology of obesity [224]. The release of proinflammatory cytokines, such as TNF-α, IL-6, and MCP-1, and increase in ROS generation occur in excessive adipose tissue [225,226]. Moreover, it has been reported that an excess of nutrients and a state of oxygen deficiency (hypoxia) stimulate adipocytes to secrete proinflammatory agents, including IL-6 and leptin, and induce oxidative stress [223].

#### Anti/Prooxidant Activity of Vitamin D

The active form of vitamin D has been reported to exert an antioxidant effect in adipocytes and adipose tissue. Vitamin D, in the form of calcitriol, was tested in vitro for its effect on the metabolism of 3T3-L1 adipocyte cells exposed to high glucose conditions. The treatment of cells with 25–50-nmol/L calcitriol resulted in decreased ROS production and the reduction of NOX4 expression. Moreover, increased expressions of *Nrf2* and antioxidant protein Trx were observed, showing that calcitriol can prevent oxidative stress by regulating the NOX4/Nrf2/Trx-signaling pathway in high-glucose-treated 3T3-L1 adipocyte cells [52].

The antioxidant effect of vitamin D has been also confirmed in human adipose tissue. VAT, obtained via abdominal surgery from patients with obesity and incubated for 12 h in the presence of 100 nmol/L calcitriol, showed decreased level of oxidative stress compared to VAT samples incubated without vitamin D [227]. The antioxidant activity of vitamin D was also found in adipose tissue obtained postmortem from rats upon the 5-week administration of vitamin D. The concentrations of antioxidant enzymes, such as GPx and SOD in adipose tissues of rats treated with HFD and vitamin D, were higher compared to HFD rat specimens without vitamin D supplementation. Moreover, the concentration of TNF-α, which is an activator of NADPH oxidase leading to ROS formation, was decreased in the adipose tissue of vitamin D-supplemented HFD rats [208]. The antioxidant activity of vitamin D was demonstrated by reducing the expression of NOX and ROS production in glucose-treated adipocytes [52] and increasing the expression of SOD and GPx in adipose tissue [208] but not by direct ROS scavenging, since 1,25(OH)_2_D did not inhibit H_2_O_2_ production [227]. However, the in vitro prooxidant activity of calcitriol was also reported. The addition of calcitriol to glucose (30 mmol/L)-treated 3T3-L1 adipocytes resulted in the stimulation of ROS production and increased NADPH oxidase expression. The stimulating effect of calcitriol on ROS production was concluded to be both due to the inhibition of mitochondrial uncoupling and increase in adipocyte intracellular Ca^2+^. Such a high concentration of glucose can generate ROS and oxidative stress. Thus, vitamin D is not able to act as an antioxidant with such high glycemia [228]. Moreover, vitamin D supplementation did not change the MDA content in the adipose tissue of HFD rats compared to rats without vitamin D administration, and the content of this oxidative stress biomarker was also at a comparable level with the tissue of normal diet rats [208].

Conflicting results from the collected literature data suggest the necessity of further investigations of the possible pro/antioxidant effects of vitamin D on adipocytes/adipose tissue. Adipocytes treated with high glucose or high free fatty acids displayed excessive ROS production and a loss of the mitochondrial membrane potential [229]. Mitochondria are the sources of ROS production, and hence, the effect of active vitamin D forms on ROS accumulation and mitochondrial dysfunction in glucose/free fatty acids-treated adipocytes should be investigated.

In vivo human studies have shown a relationship between overweight/obesity, oxidative stress, and vitamin D status. In a study of 158 youth overweight/obese, an association was found between oxidative stress, VAT accumulation, and vitamin D deficiency [230]. Another study of 47 overweight/obese patients with T2DM found that more obese patients were accompanied by increased oxidative stress (serum H_2_O_2_ concentration) and decreased serum calcidiol concentrations compared to T2DM patients with a lower degree of obesity or overweight status [231]. The results performed by our team among patients suffering from metabolic disturbances and T2DM supplemented with 2000 IU of cholecalciferol for 3 months demonstrated that vitamin D reduces the level of oxidative DNA damage. What is more, a beneficial effect was accompanied by an improvement in insulin sensitivity, as measured by a reduction of HOMA-IR and the TG/HDL ratio [232].

Vitamin D is considered as a potential remedy for metabolic diseases that are associated with a high oxidative stress level and can lead to the development of disorders such as cardiovascular diseases. The majority of conducted experimental studies indicate that the antioxidant activity of vitamin D results from its ability to influence the expression of genes involved in antioxidant defense mechanisms. However, due to the limited number of studies and a lack of data on vitamin D dosage and duration of supplementation, further studies are required to determine the effect of vitamin D administration on the antioxidant defense system and the level of oxidative stress in patients with obesity and T2DM. More specifically, population-based in vivo studies on standard blood sampling procedures and vitamin D serum concentration measurements should be broadened by adipose tissue biopsy [233,234] and meta-analyses in order to determine the direct effect of vitamin D administration on the oxidative status in adipose tissue.

### 4.8. The Effect of Vitamin D on Thermogenesis

It is long been known that thermogenesis is responsible for heat generation and the maintenance of a stable body temperature. In obesity, thermogenesis reduces fat stores through β-oxidation processes, which contributes to the production of ATP and heat. The increase in thermogenesis and fat oxidation in response to a vitamin D-rich breakfast intake showed a direct relationship between vitamin D and metabolism [235]. BAT expresses UCP1, which strengthens thermogenesis. In turn, the expression of UCP2 is downregulated by VDR in response to the calcitriol treatment of human adipocytes [236]. The vitamin D-mediated upregulation of UCP2 triggers accelerated thermogenesis in mice fed a high-calcium diet.

In contrast, Wong et al. demonstrated that VDR null mice showed a reduction in adipose tissue storage, cholesterol, and TG level at a normal calcium concentration compared to wild-type mice. Moreover, BAT presented an increased expression of UCP1, UCP2, UCP3, and β-oxidation in VDR null mice compared to wild-type mice. These data indicated that vitamin D is involved in energy metabolism and adipocyte biology in vivo, in part by the regulation of β-oxidation and UCP expression [129]. Calcitriol was reported to affect mitochondrial membrane proteins UCP-1 and UCP-2 in adipocytes [237]. The role of UCP-1 is to participate in the dissipation of energy in a form of heat (thermogenesis) [226], and the role of UCP-2 is the control of ATP synthesis and regulation of fatty acid metabolism [225]. Exposure to calcitriol increased the expression level of UCP-1 in palmitic acid-treated 3T3-L1 adipocytes [237] and decreased the expression level of UCP-2 in human adipocytes or fatty acid-stimulated human adipocytes [236].

To conclude, the number of studies focused on the findings from studies focused on the influence of vitamin D on the controlling of thermogenesis and fat storage in the body are still inconsistent and limited. The currently available data seem to show that vitamin D raises thermogenesis in mice, but further in vitro and in vivo studies are required to determine the vitamin D role in these processes.

## 5. Is Vitamin D Level Related to Lipid Metabolism Disorders and Obesity? Results from Interventional Clinical Trials

The results of most interventional clinical trials suggest a beneficial effect of vitamin D supplementation on lipid metabolism disorders and obesity. Table 2 presents selected interventional clinical trials aimed to determine the effect of vitamin D supplementation on fat mass, inflammation, insulin resistance, and the metabolic parameters connected with lipid metabolism disorders, i.e., NAFLD, T2DM, metabolic syndrome, and obesity.

One can see from Table 2 that vitamin D improves the parameters connected with lipid and carbohydrate metabolisms; insulin resistance; inflammation; and obesity (fat mass, BMI, and WHR). However, there is also data indicating that vitamin D has no effect on the clinical parameters associated with metabolic disorders. These discrepancies can be the result of the following differences in study designs between particular clinical trials. Firstly, studies’ populations come from various geographical regions, and the season of supplementation with vitamin D can directly affect endogenous vitamin D synthesis and its serum level between individual subjects. Secondly, individual features, including skin pigmentation, age, and skin grafts, also influence endogenous vitamin D synthesis. It should be noted that some studies have involved the elderly who presented a decreased capacity to synthesize the active form of vitamin D. Thirdly, there have been significant disparities between individual studies in terms of the length of supplementation, dose, and type of vitamin D. Next, we found that basal serum levels of vitamin D were diverse in individual studies. The severity of metabolic diseases in participants between particular studies, measured as the basal level of lipids, fasting plasma glucose, HbA1c, HOMA-IR, fat mass, WHR, etc., should be also taken into consideration. These limitations may significantly influence the final effect observed after supplementation with vitamin D. Finally, the response to vitamin D supplementation may have different between overweight, obese, and lean subjects due to varieties in volumetric dilution. Thus, it is extremely important to choose the appropriate dose of vitamin D for an obese person who has a low level of this vitamin in their serum but high amounts stored in adipose tissue. An interesting issue seems to be whether an excess of vitamin D stored in the adipose tissue of obese people can be released and whether it can be toxic. For these reasons, further clinical trials are needed to definitely evaluate the benefits connected with vitamin D supplementation in subjects who differ in the level of severity of metabolic disorders and obesity.

## 6. Conclusions

**Figure 5:**
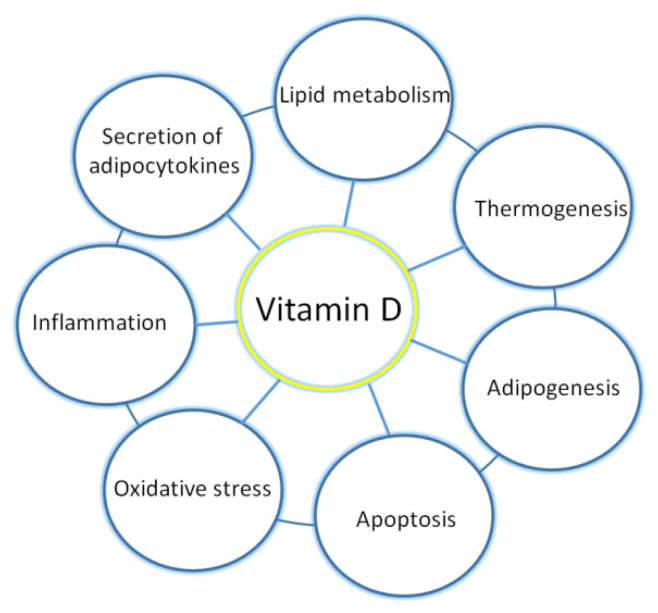
The role of vitamin D in adipose tissue. Vitamin D plays an important role regulating different signaling pathways and metabolic processes; adipogenesis, apoptosis, oxidative stress, inflammation, secretion of adipocytokines, lipid metabolism, and thermogenesis, contributing to its suitable functioning and homeostasis.

The current review presents the effect of vitamin D on adipose tissue and its clinical significance. The literature data indicate that the active form of vitamin D is produced, stored, and degraded in adipose tissue. Moreover, both VDR and 1,25D-MARRS are expressed in adipocytes, allowing vitamin D to exert a genomic and nongenomic response in adipose tissue. Vitamin D exerts an effect on adipogenesis, apoptosis, oxidative stress, inflammation, the secretion of adipocytokines, lipid metabolism, and thermogenesis, as presented in Figure 5. Thus, it contributes to the maintenance of adipose tissue structure, function, and fat content. Therefore, it is worth speculating that vitamin D supplementation may be a promising means of improving the dysfunctional adipose tissue found in T2DM and obesity. However, further clinical studies are needed to establish the benefits of vitamin D supplementation in subjects with varying levels of severity of metabolic disorders and obesity.

## Figures and Tables

**Table 1:** Natural analogs of vitamin D and their structures and sources [30,35,36,37].

Vitamin D	Structure	Synonym	Sources
D_2_	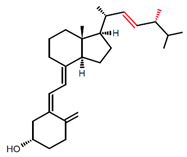	Ergocalciferol	Produced from ergosterol. Plants, fungi.
D_3_	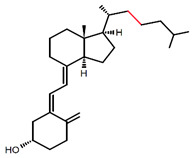	Cholecalciferol	Produced from 7-dehydrocholesterol. Fish, agriculture animals, dairy products, egg yolk, and skin of vertebrates.
D_4_	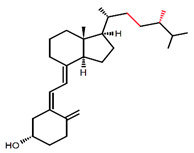	22-dihydroergocalciferol, 22,23-dihydroercalciol	Produced from 22,23-dihydroergosterol. Mushrooms.
D_5_	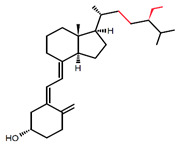	Sitocalciferol	Converted from 7-dehydrositosterol found in *Rauwolfia serpentina*

**Table 2:** The outcome of the interventional clinical trials focused on the effect of vitamin D supplementation on the metabolic parameters related to lipid metabolism. ↑—increase, ↓—decrease and ↓↑—no statistical effect.

Reference	Target Population	Study Design	Results on Studied Parameters
Sharifi et al. 2016 [238]	NAFLD Intervention group (*n* = 27: 13 men (age: 39 [31, 48.5]) and 14 women (age: 41 [35, 46.7])) received 50,000 IU of calcitriol every 14 days for four months Placebo group (*n* = 26: 13 men (age: 35 [32, 55]) and 13 women )age: 47 [43.5, 52.5]))	Double-blind, randomized-controlled clinical trial	↓TC, ↓LDL-C (in women) ↑TC, ↓↑LDL-C (in men)
Lorvand Amiri et al. 2017 [239]	NAFLD Intervention groups: - received 1000 IU of calcitriol daily for 12 weeks (*n* = 37: 22 men and 15 women; age: 39.8 ± 11) - received 500-mg calcium carbonate + 1000 IU of calcitriol daily for 12 weeks (*n* = 37: 23 men and 14 women; age: 38 ± 10.1) Placebo group (*n* = 36: 23 men and 13 women; age: 44 ± 10.8)	Randomized, placebo-controlled, double-blind clinical trial	↓WT, ↓BMI, ↓fat mass, ↓FPG, ↓insulin, ↓HOMA-IR, ↓TG, ↑HDL-C, ↓ALT
Foroughi et al. 2014 [240]	NAFLD Intervention group (*n* = 30) received 50,000 IU of vitamin D weekly over 10 weeks Placebo group (*n* = 30) 60 patients included in study (*n* = 60: 29 men and 31 women; age; 48.5)	Randomized double-blind placebo-controlled clinical trial	↓TG, ↓CRP, ↑Ca^2+^
Khosravi et al. 2018 [241]	Overweight and obese women Intervention group (*n* = 26 women; age: 29.1 ± 9.6) received vitamin D with doses 50,000 IU/w for 6 weeks Placebo group (*n* = 27 women; age: 26.9 ± 9.1)	Double-blind placebo-controlled clinical trial	↓WT, ↓WC, ↓BMI ↓↑TC, ↓↑TG, ↓↑LDL-C, ↓↑HDL-C, ↓↑FBS, ↓↑ insulin, ↓↑HOMA-IR, ↓↑WHR
Wamberg et al. 2013 [242]	Obese adults Intervention group (*n* = 26: 18 men and 18 women; age: 39.5 ± 8.0) received 7000 IU of cholecalciferol daily for 26 weeks Placebo group (*n* = 26: 7 men and 19 women; age: 41.2 ± 6.8)	Randomized double-blind placebo-controlled clinical trial	↓↑body fat, ↓↑SAT, ↓↑VAT, ↓↑IHL, ↓↑IMCL, ↓↑HOMA-IR, ↓↑blood pressure, ↓↑HDL-C, ↓↑TG, ↓↑TC, ↓↑hsCRP
Salehpour et al. 2012 [243]	Overweight and obese women Intervention group (*n* = 39 woman; age: 38 ± 7) received 1000 IU per day of cholecalciferol for 12 weeks Placebo group (*n* = 38 woman; age: 38 ± 8)	Double-blind, randomized, placebo-ontrolled, parallel group trial	↓ fat mass, ↓↑WT, ↓↑WC
Zittermann et al. 2009 [244]	Overweight subjects Intervention group (*n* = 82: 31 men and 51 women; age: 47.4 ± 10.3) received 3320 IU per day of vitamin D for 12 months Placebo group (*n* = 83: 23 men and 60 women; age: 48.8 ± 10.1)	Double-blind placebo-controlled clinical trial	↓PTH, ↓TG, ↓TNF-α, ↑LDL-C
Sneve et al. 2008 [245]	Overweight and obese subjects Intervention groups: - (*n* = 116: 57 men and 96 women; age: 46.4 ± 11.3) received calcium 500 mg/day + 40,000 IU of cholecalciferol/week for 12 months - (*n* = 106: 51 men and 92 women; age: 47.6 ± 11.9) received calcium 500 mg/day + 20,000 IU of cholecalciferol/week for 12 months Placebo group (*n* = 112: 51 men and 98 women; age: 48.9 ± 11.0) received calcium 500 mg/day + placebo for 12 months	Randomized double-blind, placebo-controlled clinical trial	↓↑WHR, ↓↑ fat, ↓↑Ca, ↓PTH
Major et al. 2007 [246]	Overweight or obese women Intervention group (*n* = 30 women; age: 43.6 ± 5.0) received 2 tablets/d of a calcium + vitamin D supplement (600-mg elemental calcium and 200 IU of vitamin D/tablet) for 15 weeks Placebo group (*n* = 33 women; age: 41.6 ± 6.1)	Double-blind, clinical placebo-controlled trial	↓LDL-C, ↓LDL:HDL ratio, ↓↑ HDL-C, ↓TG, ↓TC, ↓total HDL
Farag et al. 2019 [247]	Metabolic syndrome patients Intervention groups: - (*n* = 24: 8 men and 16 women; age:40.54 ± 5.94) received 2000 IU of vitamin D per day for 12 weeks - (*n* = 21: 7 men and 14 women; age: 40.42 ± 5.89) received 2000 IU of vitamin D per day plus 30 min of endurance physical activity for 12 weeks Placebo group (*n* = 25: 13 men and 12 women; age: 42.6 ± 5.62)	Parallel randomized placebo-controlled trial	↓TC, ↓LDL-C (in vitamin D + physical activity group) ↓↑TG, ↓↑HDL-C (in all three groups)
Mikariou et al. 2017 [248]	Metabolic syndrome patients Intervention group (*n* = 25: 15 men and 10 women; age: 52.0 ± 9.0) received 2000 IU of vitamin D/day for 3 months Placebo group (*n* = 25: 11 men and 14 women; age: 51.0 ± 12.0)	Prospective, randomized, open-label, blinded placebo-controlled end-point trial	↓↑TG, ↓↑HDL-C, ↓↑LDL-C, ↓↑FTG, ↓↑ HbA1c, ↓↑HOMA-IR, ↓↑DBP, ↓SBP
Mikariou et al. 2019 [249]	Metabolic syndrome patients Intervention group (*n* = 25: 15 men and 10 women; age: 52.0 ± 9.0)) received 2000 IU of vitamin D /day for 3 months Placebo group (*n* = 25: 11 men and 14 women; age: 51.0 ± 12.0)	Prospective, randomized, open-label, blinded placebo-controlled end-point trial	↓↑sdLDL-C, ↓↑LDL size, ↓↑LpPLA2 activity, ↓↑leptin, ↓↑ adiponectin, ↓↑leptin:adiponectin ratio
Salekzamani et al. 2016 [250]	Metabolic syndrome patients Intervention group (*n* = 35) received 50,000 IU cholecalciferol/week for 16 weeks Placebo group (*n* = 36) 71 patients included into study (*n* = 71: 35 men and 36 women; age; 40.49 ± 5.04)	Randomized placebo-controlled, double-blind parallel trial	↓TG, ↓↑FBG, ↓↑HOMA-IR, ↓↑LDL-C, ↓↑HDL-C, ↓↑TC, ↓↑WC, ↓↑BMI, ↓↑HC, ↓↑DBP, ↓↑SBP, ↓↑FP
Wongwiwatthana- nukit et al. 2013 [251]	Metabolic syndrome patients Intervention group - (*n* = 30: 16 men and 14 women; age: 62.29 ± 10.63) received 40,000 IU of vitamin D_2_ (ergocalciferol)/week for 8 weeks - (*n* = 30: 15 men and 15 women; age: 63.61 ± 13.25) received 20,000 IU of vitamin D_2_ (ergocalciferol)/week for 8 weeks Placebo group (*n* = 30: 15 men and 15 women; age: 65.07 ± 11.31)	Prospective, randomized, double-blind, Double-dummy, placebo-controlled parallel trial	↓↑FPG, ↓↑FPI, ↓↑HOMA-IR, ↓↑TC, ↓↑TG, ↓↑HDL-C, ↓↑LDL-C
Yin et al. 2016 [252]	Metabolic syndrome patients Intervention group (*n* = 61) received 700 IU of cholecalciferol/day for 12 months Placebo group (*n* = 62)	Randomized placebo-controlled intervention trial	↓↑BMI, ↓↑WC, ↓↑FPG, ↓↑FPI, ↓↑HOMA-IR, ↓↑TG, ↓↑HDL-C, ↓↑LDL-C, ↓↑SBP, ↓↑DBP
Barzegari et al. 2019 [253]	Diabetic nephropathy patients Intervention group (*n* = 25; age: 39.7 ± 7.3) received 50,000 IU of cholecalciferol/week for 8 weeks/Placebo group (*n* = 25; age: 43.7 ± 6.1)	Paralleled, randomized, double-blinded, placebo-controlled clinical trial	↓TG, ↓LDL, ↓TC, ↓↑HDL ↑↓ oxidative/antioxidative markers
El Hajj et al. 2018 [254]	Elderly subjects (nondiabetic with vitamin D deficiency) Intervention group (*n* = 60: 33 men and 27 women; age: 73.0 ± 1.95) received 30,000 IU of cholecalciferol/week for 6 months Placebo group (*n* = 55: 26 men and 29 women; age: 73.5 ± 2.1)	Randomized placebo-controlled trial	↓HOMA-IR, ↓FBG, ↓TC, ↓LDL-C, ↓BMI, ↓↑HDL-C
Tabesh et al. 2014 [255]	Nonsmoker individuals with T2DM and vitamin D insufficiency Intervention group - (*n* = 29: 15 men and 14 women; age: 50.2 ± 6.6)) received 50,000 IU of calcitriol/week for 8 weeks - (*n* = 29: 14 men and 15 women; age: 53.7 ± 5.7) received calcium 1000 mg/day for 8 weeks - (*n* = 30: 15 men and 15 women; age: 49.8 ± 6.1) received 50,000 IU of calcitriol/week + 1000 mg/day calcium for 8 weeks Placebo group (*n* = 30: 14 men and 16 women; age: 51.0 ± 6.1)	Randomized placebo-controlled clinical trial	↓serum insulin, ↓HbA1c, ↓HOMA-IR, ↓LDL-C, ↓TC/HDL-C, ↑HDL-C
Wenclewska et al. 2019 [232]	Elderly subjects with metabolic disorders Intervention group (*n* = 48: 18 subjects with T2DM and 30 controls; 14 men and 34 women; age: 63.43 ± 1.57) received 2000 IU of cholecalciferol/day for 3 months Placebo group (*n* = 44: 14 subjects with T2DM and 30 controls; 19 men and 25 women; age: 69.78 ± 2.1)	Randomized placebo-controlled clinical trial	↑HDL-C, ↓HOMA-IR, ↓TG:HDL-C ratio (in vitamin D-supplemented group) ↓HbA1c (in T2DM supplemented with vitamin D group)
Upreti et al. 2018 [256]	T2DM patients with hypovitaminosis D Intervention group (*n* = 30: 15 men and 15 women; age: 48.3 ± 9.8) received 60,000 IU of cholecalciferol/week for first 6 weeks and then once every 4 weeks until completion of the study (6 months) Placebo group (*n* = 30: 23 men and 7 women; age: 49.9 ± 6.9)	Randomized, parallel group, placebo-controlled trial	↓FPG, ↓PPPG, ↓HbA1c, ↓SBP, ↓DBP, ↓TC, ↓LDL-C, ↓↑TG, ↑↓HDL-C
Tepper et al. 2016 [257]	Healthy men without diabetes with vitamin D deficiency/insufficiency Intervention group (*n* = 78) received 100,000 IU of calcitriol/bimonthly for 12 months Placebo group (*n* = 52) 130 men included into study (*n* = 130; age; 47.52 ± 11.84)	Double-blind randomized-controlled trial	↓↑BMI, ↓↑glucose, ↓↑insulin, ↓↑hsCRP, ↓↑HOMA-IR, ↓↑HOMA-β

**Abbreviations:** TC, total cholesterol; LDL-C, low-density lipoprotein cholesterol; HDL-C, high-density lipoprotein cholesterol; PPPG, post-prandial plasma glucose; DBP, diastolic blood pressure; SBP, systolic blood pressure; BMI, body mass index; FPI, fasting plasma insulin; FPG, fasting plasma glucose; hs-CRP, high-sensitive C-Reactive Protein; TG, triglycerides; HOMA-IR, Homeostatic Model Assessment for Insulin Resistance; HbA1c, glycated hemoglobin; PTH, parathyroid hormone; FBG, fasting blood glucose; HOMA-β, Homeostatic Model Assessment of β-cells Function; TNF-α, tumor necrosis factor α; WT, weight; SAT, subcutaneous; WAT, visceral adipose tissue; IHL, intrahepatic lipids; IMCL, intramyocellular lipids; ALT, alanine aminotransferase; WC, waist circumference; FBS, fasting blood sugar; WHR, waist-to-hip ratio; FTG, fasting triglycerides; sd-LDL-C, small dense LDL-C; LpPLA2, lipoprotein-associated phospholipase A2; HC, hip circumference; FP, fat body percent. ↓—decrease, ↑—increase, and ↓↑—no statistical effect.

## Data Availability

Not applicable.
